# Enhancement of laccase activity by pre-incubation with organic solvents

**DOI:** 10.1038/s41598-019-45118-x

**Published:** 2019-07-05

**Authors:** Meng-Hsuan Wu, Meng-Chun Lin, Cheng-Chung Lee, Su-May Yu, Andrew H.-J. Wang, Tuan-Hua David Ho

**Affiliations:** 1 Institute of Plant and Microbial Biology, Academia Sinica, Taipei, 11529 Taiwan, ROC; 2 Department of Life Sciences, National Cheng Kung University, Tainan, 701 Taiwan, ROC; 3 Institute of Biological Chemistry, Academia Sinica, Taipei, 11529 Taiwan, ROC; 4 Institute of Molecular Biology, Academia Sinica, Taipei, Taiwan, ROC; 5 Agricultural Biotechnology Center, National Chung Hsing University, Taichung, 402 Taiwan, ROC; 6 Department of Life Sciences, National Chung Hsing University, Taichung, 402 Taiwan, ROC

**Keywords:** Oxidoreductases, Biocatalysis

## Abstract

Laccases that are tolerant to organic solvents are powerful bio-catalysts with broad applications in biotechnology. Most of these uses must be accomplished at high concentration of organic solvents, during which proteins undergo unfolding, thereby losing enzyme activity. Here we show that organic-solvent pre-incubation provides effective and reversible 1.5- to 4.0-fold enhancement of enzyme activity of fungal laccases. Several organic solvents, including acetone, methanol, ethanol, DMSO, and DMF had an enhancement effect among all laccases studied. The enhancement was not substrate-specific and could be observed by using both phenolic and non-phenolic substrates. Laccase preincubated with organic solvents was sensitive to high temperature but remained stable at 25 °C, for an advantage for long-term storage. The acetone-pre-incubated 3-D structure of DLac, a high-efficiency fungal laccase, was determined and confirmed that the DLac protein structure remains intact and stable at a high concentration of organic solvent. Moreover, the turnover rates of fungal laccases were improved after organic-solvent pre-incubation, with DLac showing the highest enhancement among the fungal laccases examined. Our investigation sheds light on improving fungal laccase usage under extreme conditions and extends opportunities for bioremediation, decolorization, and organic synthesis.

## Introduction

Laccases are four-copper oxidases that catalyze the oxidation of phenolic units in lignin as well as a wide range of phenolic compounds and aromatic amines^1^. Because of their oxidoreductase activity, laccases can react with 2,2′-azino-bis(3-ethylbenzothiazoline-6-sulphonic acid) (ABTS) as a substrate and as a mediator for studying laccase-mediator systems^2^. Laccase-mediator systems extend the substrate range of laccases to non-phenolic substrates and are a useful tool in organic synthesis^2,3^. Most industrially useful laccases are derived from basidiomycetes because of their high redox potential (0.8 V) as compared with ascomycete fungi, plants, and bacteria (0.4 V)^4,5^. Therefore, fungal laccases are remarkable green catalysts valuable for biotechnology and industry applications such as biodegradation^6,7^, detoxification^8^, bleaching^9^, decolorization^10,11^, organic compound synthesis^12–14^ and enzymatic biosensors^15^.

However, most of these conversions must be accomplished at high concentrations of organic solvents such as ethanol^16–18^, acetone^17,19,20^, and DMSO^17,21–23^. The use of enzymes in water-miscible organic solvents has several advantages including increased solubility of hydrophobic substrates resulting in higher yields of products^18,20,22,24^ and elimination of microbial contamination^25,26^. Accordingly, ideal fungal laccases for industrial dye-decolorization and polymerization of lignin should be highly tolerant of organic solvents^19,27,28^.

Although enzymatic catalysis in the presence of organic solvents is now a trend in biotechnology, the polar transition state of the enzyme could be destabilized by the penetration of a water-miscible organic solvent into the active site of enzyme^29,30^. Previous studies have indicated that laccases are barely tolerant of high concentrations of organic solvents because of enzyme unfolding, inactivation or denaturation^16,31–34^. Therefore, the balance between enzyme activity and substrate solubilization needs to be considered. Currently, industry and academia have devoted considerable efforts to developing effective strategies to enhance the lifetime of enzymes in organic solvents^35^. Previous studies have demonstrated an improvement in organic-solvent tolerance by encapsulation^14^, conjugation^36^, immobilization^31,37,38^, and heterologous expression of laccases in organic-solvent–tolerant microorganisms^28,34,39,40^. Novel natural laccases have been directly discovered from organic-solvent–tolerant microorganisms^41^.

This study aimed to find a technical breakthrough by combining the discovery of organic-solvent tolerance in laccases and the application of optimized reaction conditions for enhancing enzymatic catalysis in the presence of an organic solvent. We found that pre-incubation of fungal laccases with certain organic solvents significantly increased their activity toward both phenolic and non-phenolic substrates. In addition, our previously discovered *Cerrena* sp. RSD1 laccase, DLac, had the highest organic-solvent enhancement and tolerance among the fungal laccases tested^42^. The pre-incubation process also altered DLac thermostability and kinetics. We also examined the acetone-pre-incubated structure of DLac to investigate the effect of solvent molecules on the protein during pre-incubation. In summary, we developed a novel approach that could further improve laccase activity for extending its industrial use.

## Results

### Organic solvents affect DLac activity

**Figure 1:**
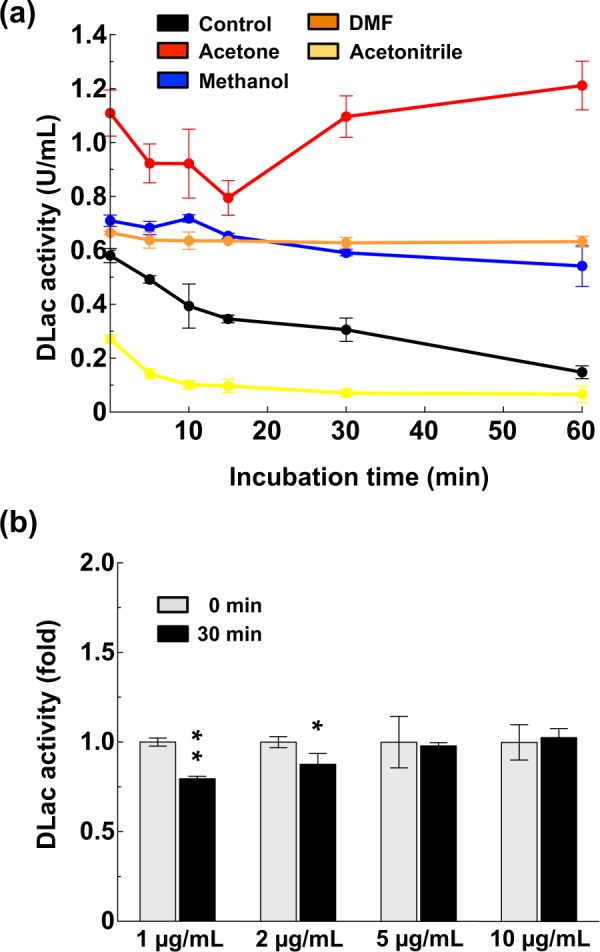
Organic solvents affect DLac activity and stability. (**a**) Effect of acetone, methanol, DMF and acetonitrile on DLac activity. The protein concentration of DLac was 1 μg/mL. (**b**) Changes in DLac activity under different protein concentrations measured with or without 30 min pre-incubation. The pre-incubation condition in this experiment is DLac incubated in Tris buffer without any organic solvent. One-fold of DLac activity was defined as the DLac activity without pre-incubation. The significant difference (*) was set at P < 0.05 and the highly significant difference (**) was set at P < 0.001 in Student’s t-test, compared to their 0 minute control. Data were shown as mean ± SD from at least three independent repeats.

We evaluated the organic-solvent effect on DLac activity and stability by pre-mixing organic solvents with 1 μg/mL DLac protein solution and then determining its enzyme activity by using the non-phenolic substrate ABTS, reported to be stable in the presence of ethanol, DMSO, and acetonitrile^39^. Immediately after the addition of 30% acetone, DLac activity was increased, whereas acetonitrile repressed the activity (Fig. 1a). Although acetone significantly enhanced DLac activity, the increment with 30% methanol and DMF was not significant. As well, DLac activity remained stable in the presence of acetone, methanol, and DMF, even after 1 hr of incubation. In contrast, pre-incubation with Tris buffer or acetonitrile decreased enzyme activity over time, with acetonitrile showing more severe inhibition (Fig. 1a). Because DLac activity decreases over time under low protein concentration (1 μg/mL), we designed our experiments for DLac remaining stable during incubation. We increased the protein concentration to ensure that DLac activity remained stable during pre-incubation. At 1 and 2 μg/mL pre-incubation, DLac activity decreased over time but remained stable at 5 and 10 μg/mL (Fig. 1b). Therefore, we used 5 μg/mL as the incubation concentration of DLac to study the enhancement effect of organic solvent pre-incubation.

### Immediate and reversible enhancement of DLac activity by organic solvent

**Figure 2:**
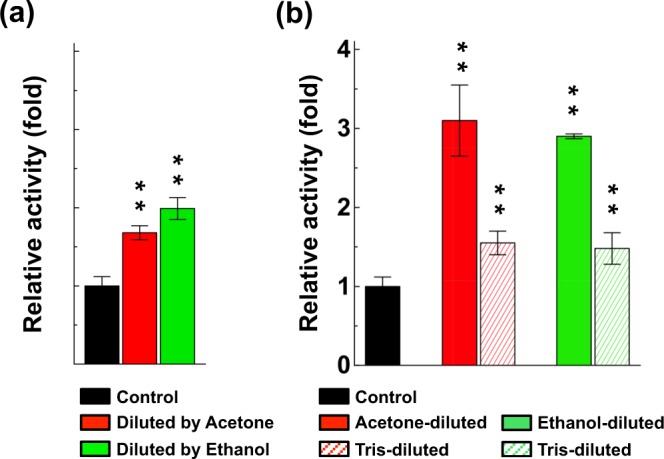
Organic-solvent enhancement is immediate and reversible. (**a**) Acetone and ethanol were used to dilute 5 μg/mL DLac into 1 μg/mL after 1 hr pre-incubation without organic solvents. (**b**) The organic-solvent enhancement of DLac activity was reversible. Acetone- or ethanol-pre-incubated with 5 μg/mL DLac was diluted to 1 μg/mL by organic-solvent–free buffer to evaluate reversibility (Tris-diluted). In comparison with reversible experiments, the corresponding organic solvent was used to dilute 5 μg/mL organic-solvent-pre-incubated DLac into 1 μg/mL (Acetone-diluted or Ethanol-diluted), respectively. One-fold of DLac activity is defined as the activity in absence of organic solvents. The significant difference (*) was set at P < 0.05 and the highly significant difference (**) was set at P < 0.001 in Student’s t-test, compared to their 0% organic solvent pre-incubation control. Data were shown as mean ± SD from at least three independent repeats.

To test whether organic solvents have rapid effects on laccase activity, we incubated DLac in organic solvent-free buffer, then diluted the DLac solution with incubation buffers containing 30% organic solvent to 1 μg/mL DLac solution before enzyme activity assay. The immediate addition of acetone was sufficient for 1.7-fold enhancing DLac activity (Fig. 2a). Besides testing the aprotic solvents acetone and acetonitrile, we also tested the immediate effect of ethanol on DLac, because ethanol is a protic solvent commonly used in industry. Ethanol had a similar 2.0-fold enhancement effect on DLac activity as acetone (Fig. 2a), which suggests that the difference between aprotic and protic solvent properties did not influence the enhancing or repressing effect on DLac activity. Because organic solvents have immediate effects on DLac activity, we wondered whether the response was reversible. Therefore, we diluted the DLac solution pre-incubated with organic solvent with organic-solvent–free buffer before the enzyme activity assay. We diluted acetone- or ethanol-pre-incubated DLac with the corresponding organic solvents as positive controls, which showed 3-fold enhancement of DLac activity (Fig. 2b). However, we observed only a slight enhancement (almost 1.5-fold) of DLac activity after dilution with organic-solvent–free buffer (Fig. 2b). Our results indicate that the enhancing effect of acetone and ethanol on DLac activity was immediate and reversible.

### Pre-incubation enhances enzyme activity of DLac under industrial conditions

**Figure 3:**
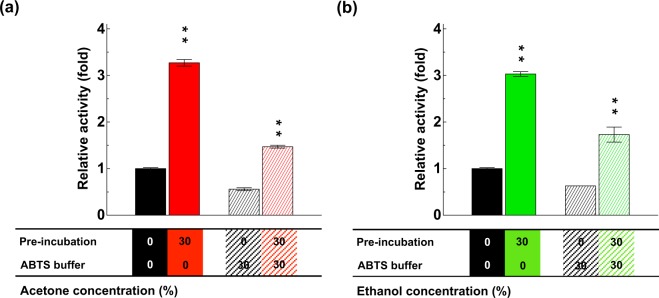
Pre-incubation has advantages on enhancing DLac activity in the presence of the corresponding organic solvent. The organic-solvent tolerance of enzyme activity of DLac was investigated by adding acetone (**a**) or ethanol (**b**) into ABTS enzyme assay buffer. The 0% acetone (or 0% ethanol) pre-incubation condition in this experiment was laccase incubated in Tris buffer without any organic solvent. DLac activity without organic solvents during 1 hr pre-incubation and ABTS enzyme assay was defined as 1-fold. The significant difference (*) was set at P < 0.05 and the highly significant difference (**) was set at P < 0.001 in Student’s t-test, compared to their 0% organic solvent pre-incubation control. Data were shown as mean ± SD from at least three independent repeats.

Since the organic solvents are normally mixed with substrates for industrial purposes, we needed to evaluate the organic-solvent adaptation of DLac activity during enzyme catalysis. We added organic solvent into substrate buffer and measured DLac activity. In contrast to pre-incubation results, the addition of acetone or ethanol into substrate buffer decreased DLac activity (Fig. 3a,b). Regardless, DLac showed 56% and 63% residual activity with acetone and ethanol, respectively. The residual activity was as high as R2 laccase, which by far showed the best organic-solvent tolerance: 60% of residual activity in 30% ethanol^34^. In contrast, most of the reported fungal laccases from *Trametes hirsuta*, *Trametes versicolor*, *Myceliophthora thermophile*, *Pycnoporus cinnabarinus*, *Coriolopsis gallica* and *Pleurotus ostreatus* retained only 10% activity under the same condition^34^. Although the presence of organic solvent in the substrate buffer reduced DLac activity, DLac pre-incubated with organic solvent still showed 3-fold higher enzyme activity. Pre-incubating DLac with acetone or ethanol could enhance and maintain the enzyme’s catalysis efficiency to substrate mixed with the same organic solvent.

### Acetone sensitizes DLac to high temperature

**Figure 4:**
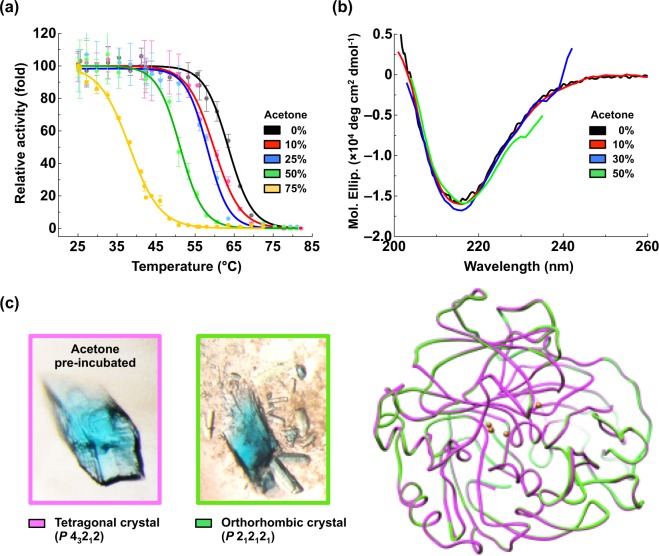
Thermostability and 3-D structure of acetone-pre-incubated DLac. (**a**) Thermostability of DLac was decreased with increasing acetone concentration. Plots indicated the residual activities after 10 min of heat treatment. DLac activity after 10-min incubation at 25 °C was defined as 100%. Data were shown as mean ± SD from at least three independent repeats. (**b**) The secondary structure of DLac remained unchanged in acetone solution. Far-UV CD spectra were used to monitor DLac structure change in 0, 10, 30 and 50% acetone. (**c**) Crystals and 3-D structures of the tetragonal form of acetone-pre-incubated DLac and orthorhombic form of DLac were compared and superimposed. Four copper atoms are represented as orange spheres.

Because organic-solvent pre-incubation increased DLac activity (Fig. 1a) and knowing that temperature is a critical factor regulating enzyme stability, folding and activity, we investigated the thermostability of DLac in the presence of acetone. The thermostability of DLac decreased with increasing acetone concentration, with T_50_ at 63.5 °C, 59.8 °C, 58.1 °C, 51.2 °C and 38.2 °C on incubation with 0%, 10%, 25%, 50% and 75% acetone, respectively (Fig. 4a). Thus, DLac pre-incubated with organic solvent became more sensitive to high temperature and lost its activity rapidly with acetone content above 50%.

### Stable 3-D structure of DLac in high concentration of acetone

To monitor any structural change in DLac during organic-solvent pre-incubation, we used circular dichroism (CD) to observe the difference in secondary structure of DLac under different acetone concentrations. Surprisingly, even at 50% acetone, the secondary structure of DLac was unchanged (Fig. 4b). The signal between 230 and 240 nm looks truncated because their high tension (HT) voltages were too high to reveal reliable CD spectra at 30 and 50% acetone solution. Stepankova *et al*. also reported CD spectra were limited between 200 to 235 nm when using 20 to 40% acetone solution, indicating that high concentrations of acetone may interfere CD signal^43^. Previous studies from Li *et al*. and Kelly *et al*. suggested that a reasonable HT reading should be less than 700 V without reducing the signal-to-noise ratio^44,45^. Therefore, when the Far-UV CD spectra were used to monitor DLac structure change in Fig. 4b, we also measured its HT voltage (Supplementary Fig. S1) to make sure the data is reliable. We also crystalized acetone-pre-incubated DLac to investigate whether the protein was modified or bonded by acetone molecules. DLac after 1-day acetone-pre-incubation was prepared for crystallization. The crystallization drop was consisted of 1 μL protein solution containing 30% acetone and 1 μL reservoir solution, and was crystallized at a *P*4_3_2_1_2 space group (tetragonal crystal) at 1.5 Å resolution (PDB code: 5Z22, Fig. 4c and Supplementary Table S1). However, this tetragonal crystal structure showed neither binding of the acetone molecule nor any chemical modification. This structure was further compared to its orthorhombic crystal form^42^ (PDB code: 5Z1X) that was observed in absence of actone. Two crystal structures could be perfectly superimposed with an overall RMSD of 0.143 Å for 494 C atoms (Fig. 4c). At the crystallization stage, the crystal-growth time was 2, 9 and 20 days with pre-incubated acetone concentration 30%, 20% and 0%, respectively, which suggests that acetone may accelerate DLac crystallization. The space group was changed to *P*4_3_2_1_2, different from the orthorhombic form of *P*2_1_2_1_2_1_ (Fig. 4c). Both protein crystals were blue from the Type 1 copper of the DLac (Fig. 4c). Notably, CD and crystal structure analysis indicated that even at high acetone concentration, the 3-D structure of DLac was not damaged. Thus, this immediately reversible enhancement was due to neither chemical modification nor covalent binding with solvent molecules.

### Organic solvent effects are general across fungal laccases

**Figure 5:**
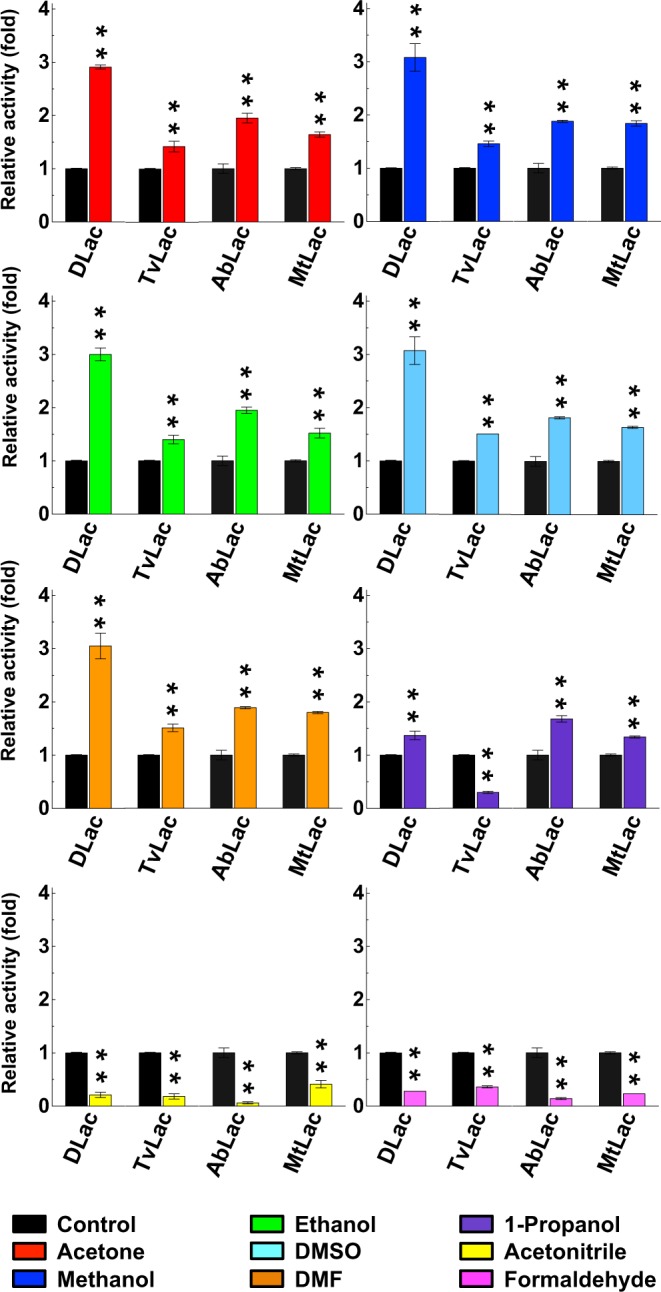
Organic-solvent enhancement of enzyme activity of general fungal laccases. Four purified laccases were pre-incubated with eight different solvents, and after 1 hr of pre-incubation, enzyme activity was measured by enzyme activity assay with ABTS used as a substrate. The enzyme activity of each laccase without organic solvent during pre-incubation was defined as 1-fold. The highly significant difference (**) was set at P < 0.001 in Student’s t-test, compared to their Tris buffer control.

To confirm that the acetone enhancement phenomenon we found is general across fungal laccases, we included commercial fungal laccases from *T. versicolor* (TvLac), *Agaricus bisporus* (AbLac) and *M. thermophila* (MtLac). Our phylogenetic analysis based on amino acid sequence identity indicated that these four laccases were dispersed among ascomycetes and basidiomycetes (Supplementary Fig. S2). These laccases were purified and incubated at 5 μg/mL (Supplementary Fig. S3). All tested laccases at this protein concentration remained stable with constant laccase activity in Tris buffer (as a control condition) during preincubation (Supplementary Fig. S4). Therefore, we used the 5-μg/mL concentration for testing the effect of organic solvents on laccase activity. We pre-incubated laccases with eight different water-miscible solvents commonly used in industry, including alcohols (methanol, ethanol and 1-propanol), ketones (acetone and DMSO), DMF, acetonitrile and formaldehyde. All tested laccases showed enhanced activities after pre-incubation with acetone, methanol, ethanol, DMSO and DMF (Fig. 5 and Supplementary Fig. S5). DLac activity was enhanced by nearly 3-fold, with a 1.5- to 2-fold enhancement for TvLac, AbLac and MtLac. In contrast to methanol and ethanol pre-incubation, 1-propanol pre-incubation only slightly enhanced DLac, AbLac and MtLac activity and failed to enhance TvLac activity. In addition, acetonitrile and formaldehyde pre-incubation severely reduced laccase activity.

**Figure 6:**
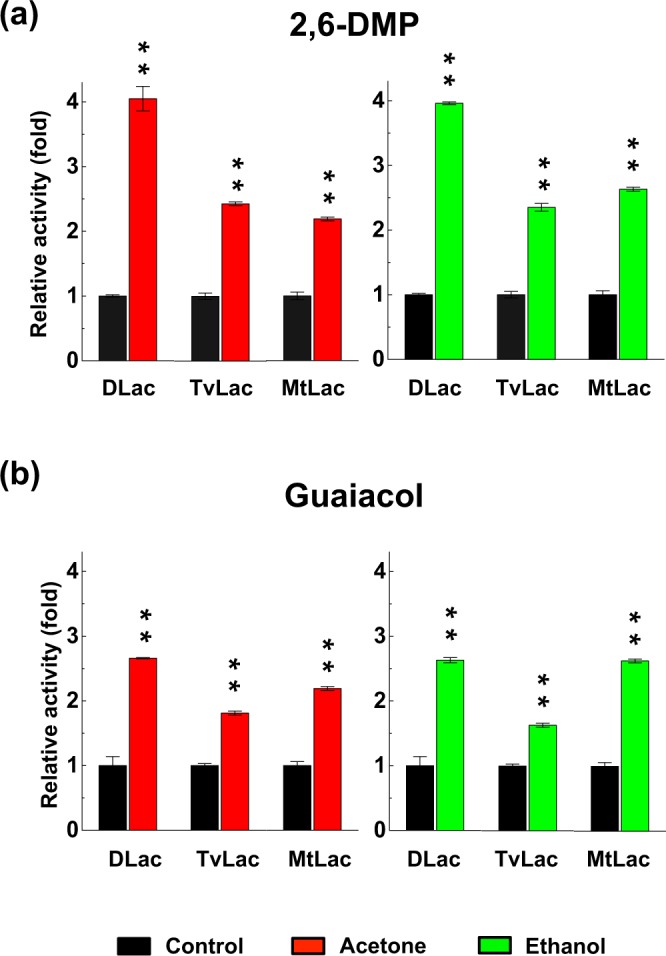
The enhancement effect of organic-solvent pre-incubation on enzyme activity observed by using phenolic substrates. Acetone and ethanol pre-incubation enhanced laccase activity with 2,6-DMP (**a**) or guaiacol (**b**) used as phenolic substrates. Purified laccases including DLac, TvLac, and MtLac were pre-incubated with acetone or ethanol for 1 hr before enzyme activity assay. The enzyme activity of each laccase without organic solvent during pre-incubation was defined as 1-fold. The highly significant difference (**) was set at P < 0.001 in Student’s t-test, compared to their Tris buffer control.

To test whether the enhancement was substrate-specific, we used two different phenolic substrates, 2,6-DMP (Fig. 6a and Supplementary Fig. S6a) and guaiacol (Fig. 6b and Supplementary Fig. S6b), after acetone and ethanol pre-incubation. DLac, TvLac and MtLac showed enhanced activities after incubation, whereas AbLac showed no activity toward 2,6-DMP and guaiacol (Supplementary Figs S5a and S5b). Among the three different substrates, DLac conferred the highest increase (4-fold) in activity when oxidizing 2,6-DMP, whereas TvLac and MtLac conferred only a 2.2- to 2.4-fold increase. When using guaiacol as the substrate, DLac and MtLac conferred a similar and higher fold change (2.2- to 2.7-fold) in activity as compared with TvLac (1.6- to 1.8-fold). Although phylogenetic analysis separated DLac and MtLac into different groups (Supplementary Fig. S2), the effect of organic-solvent pre-incubation on enzyme activity was similar between these fungal laccases (Fig. 6b). Our results suggest that acetone enhancement of fungal laccase activity is not substrate-specific.

### Acetone pre-incubation improved turnover rate and catalytic efficiency

**Figure 7:**
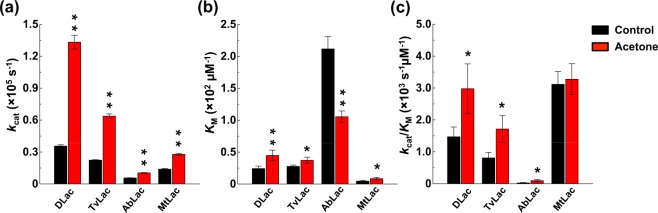
Acetone pre-incubation improve turnover rate and catalytic efficiency. The turnover rate (*k*_cat_, **a**), substrate-binding affinity (*K*_M_, **b**) and catalytic efficiency (*k*_cat_/*K*_M_, **c**) of laccases without or with acetone were measured. Four purified laccases were pre-incubated with acetone, and their enzyme activity was immediately measured by enzyme activity assay with ABTS used as a substrate at 30 °C. The significant difference (*) was set at P < 0.05 and the highly significant difference (**) was set at P < 0.001 in Student’s t-test, compared to their Tris buffer control.

Because we observed the enhancement effect of acetone on enzyme activity, we then used kinetic analysis of fungal laccases in the absence or presence of acetone (Fig. 7 and Table 1). In the presence of acetone, the turnover rate of DLac, TvLac, AbLac and MtLac was 3.7-, 2.8-, 1.8- and 2.0-fold increased, respectively, as compared with the non-acetone condition (Fig. 7a and Table 1). Most of the laccases except AbLac showed increased *K*_*M*_ in the presence of acetone (Fig. 7b and Table 1), which suggests that substrate affinity was decreased after acetone pre-incubation. This observation is consistent with previous findings of only AbLac having different substrate preference among the laccases tested (Supplementary Figs S6a and S6b), and the turnover rate of AbLac did not reach the diffusion limit. Nevertheless, all of the acetone-pre-incubated laccases showed increased catalytic efficiency (*k*_cat_/*K*_M_, Fig. 7c and Table 1). Among basidiomycete laccases, (DLac, TvLac and AbLac), the catalytic efficiency was increased in the range of 1.99- to 3.77-fold. However, the ascomycete MtLac, located farthest from other basidiomycete laccases in the phylogenetic tree (Supplementary Fig. S2), showed only 1.05-fold increase in catalytic efficiency. In summary, all laccases belonging to diffusion-limited enzymes had a higher *k*_cat_, *K*_M_ and *k*_cat_/*K*_M_ value. Thus, acetone pre-incubation may improve the turnover rate and catalytic efficiency of laccases.

**Table 1:** Kinetic analysis of fungal laccases.

	Acetone conc. (v/v,%)	*k*_cat_ (10^4^ s^−1^)	*K*_M_ (μM)	*k*_cat_/*K*_M_ (10^3^ s^−1^ μM^−1^)
DLac	0	3.56 ± 0.12	24 ± 3.9	1.47 ± 0.31
	30	13.33 ± 0.65	45 ± 8.3	2.98 ± 0.78
TvLac	0	2.24 ± 0.04	29 ± 2.1	0.81 ± 0.17
	30	6.38 ± 0.20	33 ± 4.8	1.72 ± 0.42
AbLac	0	0.56 ± 0.02	212 ± 19.5	0.03 ± 0.01
	30	1.04 ± 0.03	105 ± 9.0	0.10 ± 0.04
MtLac	0	1.39 ± 0.05	4 ± 1.2	3.12 ± 0.40
	30	2.78 ± 0.09	8 ± 1.9	3.28 ± 0.49

## Discussion

The addition of organic solvents significantly changes enzyme activity and stability. Several enzymes, including PST-01 protease and subtilisin, have a longer half-life in solution containing methanol, ethanol, acetone, DMF, or DMSO than without organic solvent^46^. In our study, DLac also remained stable in the presence of acetone, methanol, and DMF (Fig. 1a). To verify the potential of using laccase in the presence of organic solvents, studies were ordinarily evaluated by adding organic solvents into the enzyme assay solution. Several fungal laccases from ascomycetes (*M. thermophila* and *Thielavia* sp.) and basidiomycetes (*T. versicolor*, *A. bisporus*, *P. ostreatus*, *P. cinnabarinus*, *Cerrena unicolor* and *C. gallica* etc.) showed lower enzyme activities in the presence of ethanol, acetone, acetonitrile or DMSO^24,34,47^. DLac seemed to be less sensitive to the organic-solvent inactivation and retained nearly 60% of its activity (Fig 3a,b). In addition to its organic-solvent tolerance, DLac showed the highest activity enhancement (3.0-fold) after pre-incubation with acetone, methanol, ethanol, DMSO, and DMF (Figs 2b, 3a,b and 5). This organic-solvent–induced enzyme activity enhancement has been observed for other enzymes such as elastase strain K (1.2-fold)^48^ and EMB9 protease (1.5-fold)^49^. Solvent pre-incubated DLac showed 3-fold higher laccase activity compared to DLac incubated with the Tris buffer control, regardless the organic solvent was mixed with or without the substrate (Fig. 3a,b).

One of the intriguing properties of organic-solvent enhancement is the reversibility when the organic solvent is substituted by Tris buffer (Fig. 2b). This offset of organic-solvent enhancement is similar to previous study of “molecular memory” effects and high protein conformational rigidity observed in the presence of organic solvent^50^. In comparing the enhancement effects of alcohols among four different fungal laccases, propanol with the longest hydrocarbon chain generated relative lower enhancement, whereas methanol and ethanol provided higher-fold enhancement (Fig. 5). This phenomenon agreed with the previous organic-solvent tolerance study of bacterial laccase showing that alcohols with short hydrocarbon chains could conserve the enzyme stability and activity^51^. In contrast, acetonitrile always severely lowered laccase activities (Figs 1a and 5), which is consistent with a previous organic-solvent tolerance study of TvLac and recombinant MtLac expressed in *Saccharomyces cerevisiae*^39^. Both high redox potential basidiomycete (DLac, TvLac, and AbLac) laccases and low redox potential ascomycete (MtLac) laccases show similar organic-solvent enhancement of their activities (Fig. 5). Our results suggest that organic-solvent enhancement is general for fungal laccases and is not related to fungal class or redox potential.

Protein unfolding can be induced by organic solvents^30^, resulting in the loss of function due to the direct contact between the protein and organic solvents^48,52,53^. This study provided 3-D structure evidence to confirm that DLac maintains its protein structure at high acetone concentration (Fig. 4c). The structural study of 6B lipase and elastase strain K, showing high enzyme activity in the presence of organic solvents, indicated that active-site conformation and protein core should be maintained^48,53^. Our structure analysis indicated that high acetone concentration did not destroy the overall 3-D structure of DLac. The presence of organic solvents may improve enzymatic turnover rate by causing microenvironmental changes surrounding the flexible regions that form a substrate-binding pocket. The substrate-binding pocket of DLac consists of hydrophobic residues involved in the formation of activated form of this enzyme. In a recent study, Gu *et al*. conducted molecular dynamics simulations to investigate how methanol molecules were able to enter into the active site of an organic solvent-stable protease, leading to microenvironmental changes around the aromatic amino acids. Their results suggest that high concentrations of methanol lead to the exposure of hydrophobic groups to the solvent, leading to decrease in enzyme activity and stability^54^. Since aromatic and hydrophobic amino acids are very sensitive to organic solvents, Loo *et al*. determined the chemical shifts in 2D [^15^N, ^1^H]-heteronuclear single quantum coherence spectroscopy (HSQC) and suggested that organic solvent molecules were preferentially localized to hydrophobic regions near the enzymatic active site. Interestingly, the enzyme activity increased under low concentrations of DMSO, isopropanol and methanol, but decreased in the presence of acetone, acetonitrile and DMF^53^. Moreover, studies of enzyme suspensions in anhydrous solvents also indicated that the addition of a small amount of water to the anhydrous solution significantly enhanced the enzyme activity of subtilisin, alcohol oxidase, polyphenol oxidase, and alcohol dehydrogenase^50,55,56^. These relevant studies indicated the microenvironment changes caused by organic solvents may be pivotal for the regulation of enzyme activity. The molecular dynamics simulation/determination and electrospray-ionization (ESI) mass spectrometry could be used to more effectively reveal the mechanism underlying the phenomenon described in this work, which could be a good topic for future studies^54,57^.

To the best of our knowledge of diffusion-limited laccases, the broader substrate-binding cavity facilitates the diffusion rate of substrates, as reflected by a higher *k*_cat_ value^42,58^. Here, DLac, with a broader substrate-binding cavity, showed higher organic-solvent enhancement than TvLac (3-fold vs 1.5-fold, Fig. 5). Thus, basidiomycete laccases with a broad substrate-binding cavity may be highly responsive to organic-solvent enhancement. Because organic solvents are frequently used in wide industrial applications, the enhancement and maintenance of laccase activity will significantly decrease the cost of the biocatalytic procedure. This organic-solvent enhancement could increase its economic efficiency with current immobilization techniques.

## Methods

### Enzyme activity analysis

Standard laccase activity was determined by spectrophotometric measurements with the Monochromator-Based Multi-Mode Microplate Reader (BioTek). The standard reaction mixture contained 195 μL of 1 mM ABTS (*ε*_420_ = 3.60 × 10^4^ M^−1^ cm^−1^) in substrate buffer (80 mM citric acid; 40 mM Na_2_HPO_4_, pH 3.0) and 5 μL of enzyme solution. For phenolic substrate catalysis, the reaction mixture contained 195 μL of 5 mM 2,6-dimethoxyphenol (2,6-DMP, *ε*_477_ = 1.48 × 10^4^ M^−1^ cm^−1^) or 6 mM guaiacol (*ε*_465_ = 1.2 × 10^4^ M^−1^ cm^−1^) in substrate buffer (60 mM citric acid, 77 mM Na_2_HPO_4_, pH 4.0) and 5 μL enzyme solution. The enzyme assay was immediately performed at 30 °C after mixing the enzyme solution and substrate buffer. Laccase activity was calculated from the optical density (OD) changes at linear range (between 9 and 12 min), with 420, 477 and 465 nm for ABTS, 2,6-DMP and guaiacol, respectively. One unit of enzyme activity was defined as 1 μmol substrate transformed to product per minute^59^. The kinetic study of DLac involved use of 1.25–800 μM ABTS at pH 3.0, 3.13–2,000 μM 2,6-DMP at pH 4.0 and 18.78–3,000 μM guaiacol at pH 4.0. The initial rates were acquired from the linear portion of the experimental curve, and the kinetic parameters were determined by using the Michaelis-Menten model in SigmaPlot 10.0. The kinetics data were shown as mean ± standard deviation (SD) from three independent replicates.

### Purification of laccases

The production and purification of DLac was described in a previous study^42^. Three commercial laccases including *Trametes versicolor* laccase (TvLac, #38429), *Agaricus bisporus* laccase (AbLac, #40452) and recombinant *Myceliophthora thermophla* laccase (MtLac, #SAE0050) were purchased from Sigma-Aldrich. All commercial laccases were dialyzed in 20 mM Tris-HCl (pH 8.0) and purified by using a HiTrap Q column Vivaspin Turbo 15 with 30,000 MWCO and Vivaspin 500 with membrane 50,000 MWCO concentrators (Sartorius Sedium Biotech). All laccases were stored in 20 mM Tris-HCl with 150 mM NaCl (pH 8.0). The purity of the protein was verified by SDS-PAGE (Supplementary Fig. S2).

### Effect of organic solvents on laccase activity

The organic solvent pre-incubation procedure consisted of 3 steps. 1) Purified laccases (5 μg/mL) were pre-incubated in 4 mM Tris, 30 mM NaCl with 30% (v/v) of each organic solvent, including acetone, methanol, ethanol, DMSO, DMF, 1-propanol, acetonitrile and formaldehyde, at 25 °C for 1 hr. 2) The samples were diluted to a proper protein concentration by using the same buffer at the pre-incubation step or in 4 mM Tris, 30 mM NaCl for testing the reversibility of laccases. 3) The enzyme activities were measured as described in the Enzyme activity analysis section. The tolerance of laccase activity was evaluated by adding 30% organic solvent into substrate buffer (1 mM ABTS; 80 mM citric acid; 40 mM Na_2_HPO_4_, pH 3.0). All chemicals were purchased from Sigma-Aldrich. The kinetics data were shown as mean ± SD from three independent replicates.

### Thermostability

The *T*_50_ value was assessed in a gradient thermocycler (Biometra T Gradient Thermocycler), corresponding to the temperature at which the enzyme retains 50% of its activity after a 10-min incubation. Subsequently, 176 μL aliquots were used in triplicate assays at temperatures between 25 °C and 82 °C. After 10 min of incubation, the samples were immediately chilled on ice water for 10 min. An amount of 2 μL of each protein sample (5 μg/mL) was used to measure the enzyme activity, as described in the Enzyme activity analysis section. The kinetics data were shown as mean ± SD from three independent replicates.

### Far-UV circular dichroism (CD) spectropolarimetry

CD experiments were performed on a Jasco-815 spectropolarimeter equipped with a six-position Peltier temperature control system (PTC-4245). The sample was prepared with 4.73 μM DLac in the presence of 0, 10, 30 and 50% acetone in 4 mM Tris-HCl and 30 mM NaCl buffer (pH 8.0). The CD spectra were measured between 200 and 260 nm in a 1.0-mm light path length quartz cuvette with a step size of 1.0 nm, with the high tension voltage threshold set at 700 V to make sure that the signal-to-noise ratio was acceptable.

### Crystallization and data collection

For the crystallization, the protein solution was pre-incubated in 4 mM Tris-HCl and 30 mM NaCl buffer (pH 8.0) containing 30% acetone for 24 hr. The reservoir solution for protein crystallization used in this study was 1.6 M ammonium sulfate, pH 8.4. Crystals of the acetone-pre-incubated DLac were grown by mixing 1 μL protein (15 mg/mL) with 1 μL reservoir solution by using the sitting-drop vapor diffusion method at 20 °C. All crystals were flash-frozen with 20% to 25% glycerol as a cryoprotectant and the diffraction patterns were recorded at cryogenic temperatures. The diffraction data were collected at wavelength 1.000 Å by using the synchrotron TPS-05A beamline of NSRRC in Taiwan, with a Rayonix MX300-HS detector. The diffraction data were processed and scaled by using the program HKL2000^60^.

### Structure determination and refinement

The acetone-pre-incubated DLac crystal structure was determined by molecular replacement with the program MOLREP of the CCP4 program suite^61^, with the crystal structure of DLac (PDB ID: 5Z1X) used as a search model^45^. Throughout the refinement, 5% of randomly selected data were set aside for cross validation with the *R*_free_ values. The models were manually modified by using the program Coot^62^. Difference Fourier (*F*_o_-*F*_c_) maps were calculated to locate the solvent molecules. The crystal structure was refined by using Refmac5^63^, including the individual isotropic B-factor refinement. The data collection and final model statistics are shown in Supplementary Table S1. The molecular figures were produced using UCSF Chimera^46^. The atomic coordinates and structure factors of the DLac crystal structure have been deposited in the PDB (ID: 5Z22).

## Supplementary information

Supplementary Information
