# Supplementary material for: Enhancement of laccase activity by pre-incubation with organic solvents
Source: Sci Rep. 2019 Jul 5;9:9754. doi: 10.1038/s41598-019-45118-x (PMC6611822; doi:10.1038/s41598-019-45118-x)
Supplement: Supplementary file 1 — Supplementary Information [file 41598_2019_45118_MOESM1_ESM.docx]

**Enhancement of laccase activity by pre-incubation with organic solvents**

Meng-Hsuan Wu^1,2,+^, Meng-Chun Lin^1,+^, Cheng-Chung Lee^3^, Su-May Yu^4,5,6^, Andrew H.-J. Wang^3^ and Tuan-Hua David Ho^1,5,6,*^

^1^Institute of Plant and Microbial Biology, Academia Sinica, Taipei, 115, Taiwan, ROC

^2^Department of Life Sciences, National Cheng Kung University, Tainan, 701, Taiwan, ROC

^3^Institute of Biological Chemistry, Academia Sinica, Taipei, 11529, Taiwan, ROC

^4^Institute of Molecular Biology, Academia Sinica, Taipei, Taiwan, ROC

^5^Agricultural Biotechnology Center, National Chung Hsing University, Taichung 402, Taiwan, ROC.

^6^Department of Life Sciences, National Chung Hsing University, Taichung 402, Taiwan, ROC.

^*^Corresponding author:

Tuan-Hua David Ho

Phone: 886-2-2787-1079

FAX: 886-2-2782-1605

E-mail: tho@gate.sinica.edu.tw; [ho@biology2.wustl.edu](mailto:ho@biology2.wustl.edu)

^+^These authors contributed equally to this work and are considered co-first authors.

**Table S1. Data collection and refinement statistics^＊^.**

| Protein | DLac |
| --- | --- |
| Data collection |  |
| Wavelength (Å) | 1.0 |
| Space group | *P* 4_3_2_1_2 |
| Cell dimensions (Å) | *a* =87.41, *b* = 87.41, *c* =148.79 |
| Resolution (Å) | 30.0-1.50 (1.55-1.50) |
| Unique reflections | 89,774 |
| *R*_merge_ (%) | 6.4 (63.0) |
| *I*/σ(*I*) | 27.5 (1.9) |
| Completeness | 96.8 (98.1) |
| Redundancy | 4.7 (3.9) |
|  |  |
| Refinement |  |
| Resolution (Å) | 30.0-1.50 |
| No. of reflections *R*_work_/*R*_free_ | 85,148/4,525 |
| *R*_work_/*R*_free_ | 12.6/15.2 |
| No. of atoms/Avg B factor (Å^2^) |  |
| Protein | 3,739/19.3 |
| Glycan | 42/31.9 |
| Copper ion | 4/16.6 |
| Other | 602/34.3 |
| RMSD |  |
| Bond lengths (Å) | 0.007 |
| Bond angles (°) | 1.34 |
| Ramachandran statistics (%) ^＊＊^ |  |
| Ramachandran favored | 97.97 |
| Ramachandran outliers | 0.41 |

**^＊^** Values corresponding to the highest resolution shell are shown in parentheses.

**^＊＊^** The stereochemistry of the model was validated by using MolProbity^1^.

**Table S2. List of accession numbers for fungal laccases.**

| Fungal species | Accession number |
| --- | --- |
| *Agaricus bisporus* | AAC18877.1 |
| *Armillaria gallica* | PBK92547.1 |
| *Armillaria gallica* | PBK92547.1 |
| *Aureobasidium pullulans* | AUW14931.1 |
| *Auricularia auricula-judae* | AHZ58334.1 |
| *Auricularia subglabra* | EJD50415.1 |
| *basidiomycete PM1* | CAA78144.1 |
| *Botrytis Aclada* | 3SQR_A |
| *Ceratocystis platani* | KKF96573.1 |
| *Cerrena Maxima* | 3DIV_A |
| *Chaetomium globosum* | XP_001228806.1 |
| *Coemansia reversa* | PIA13334.1 |
| *Coniochaeta hoffmannii* | AUS45857.1 |
| *Coniochaeta ligniaria* | OIW22748.1 |
| *Coriolopsis gallica* | APW29214.1 |
| *Corynespora cassiicola* | PSN66585.1 |
| *Cryptococcus gattii* | ACB05902.1 |
| *Cryptococcus neoformans* | ALI93836.1 |
| *Curvularia lunata* | AFH53063.1 |
| *Curvularia lunata* | AFH53063.1 |
| *Cyathus bulleri* | ARD69912.1 |
| *Diaporthe liquidambaris* | AGZ62514.1 |
| *Dichomitus squalens* | XP_007369227.1 |
| *Didymosphaeria variabile* | AFG30951.1 |
| *Diplodia seriata* | OMP88167.1 |
| *Exidia glandulosa* | KZV96355.1 |
| *Fomitiporia mediterranea* | EJC99977.1 |
| *Fusarium culmorum* | PTD06345.1 |
| *Ganoderma lucidum* | AHA83595.1 |
| *Gloeophyllum trabeum* | EPQ52617.1 |
| *Hericium coralloides* | BAQ25793.1 |
| *Histoplasma capsulatum* | EGC46581.1 |
| *Hypsizygus marmoreus* | KYQ44884.1 |
| *Jaapia argillacea* | KDQ59157.1 |
| *Lentinula edodes* | BAJ12090.1 |
| *Lentinus sajor-caju* | CAD45379.1 |
| *Lentinus Tigrinus* | 2QT6_A |
| *Lenzites gibbosa* | AEQ28164.1 |
| *Leucoagaricus* sp. | KXN81384.1 |
| *Madurella mycetomatis* | KXX77750.1 |
| *Magnaporthe oryzae* | XP_003718807.1 |
| *Marasmius cladophyllus* | AMQ22747.1 |
| *Marssonina coronariae* | OWO97437.1 |
| *Melanocarpus Albomyces* | 1GW0_A |
| *Meripilus giganteus* | CBV46340.1 |
| *Monilinia fructigena* | ABM21605.1 |
| *Mucor ambiguus* | GAN09683.1 |
| *Myceliophthora thermophila* | XP_003663741.1 |
| *Mycena chlorophos* | GAT46418.1 |
| *Nannizzia gypsea* | XP_003169173.1 |
| *Neohortaea acidophila* | AAY33971.2 |
| *Neurospora crassa* | XP_956939.1 |
| *Ophiostoma piceae* | EPE07365.1 |
| *Panus rudis* | AAW28932.1 |
| *Phialocephala scopiformis* | XP_018071716.1 |
| *Phlebia tremellosa* | CAK54346.1 |
| *Piloderma croceum* | KIM83705.1 |
| *Pleurotus ostreatus* | CAC69853.1 |
| *Polyporus brumalis* | ABN13591.1 |
| *Punctularia strigosozonata* | EIN06931.1 |
| *Pycnoporus Cinnabarinus* | 2XYB_A |
| *Rasamsonia emersonii* | XP_013330722.1 |
| *Rigidoporus microporus* | AAO38869.1 |
| *Scedosporium apiospermum* | KEZ39002.1 |
| *Sclerotinia minor* | ABM21603.1 |
| *Serendipita vermifera* | KIM25773.1 |
| *Serpula lacrymans* | EGO25067.1 |
| *Shiraia* sp. | AHN82367.1 |
| *Sistotremastrum suecicum* | KZT40911.1 |
| *Sordaria fimicola* | AIW52362.1 |
| *Spongipellis* sp. | BAE79811.1 |
| *Sporothrix brasiliensis* | KIH89388.1 |
| *Steccherinum murashkinskyi* | AFI41888.1 |
| *Stereum hirsutum* | XP_007311222.1 |
| *Talaromyces islandicus* | CRG82760.1 |
| *Termitomyces* sp. | BAE53769.1 |
| *Thanatephorus cucumeris* | Q02081.1 |
| *Thielavia Arenaria* | 3PPS_A |
| *Trametes versicolor* | 1GYC_A |
| *Trichoderma reesei* | ETS00495.1 |
| *Trichophyton violaceum* | OAL71549.1 |
| *Valsa mali* | KUI63476.1 |
| *Verticillium dahliae* | EGY13507.1 |

**Figure S1. HT voltage was used to evaluate the reliability of Far-UV CD spectra in 0, 10, 30 and 50% acetone.** The orange dash line indicated the HT voltage threshold set at 700 V to make sure that the signal-to-noise ratio was acceptable.

**Figure S2. Distribution of four laccases in this study among fungal laccases from NCBI Protein Database.** The amino acid sequences were aligned by using Clustal W, and phylogenetic evolutionary analyses involved using MEGA 6.06 with the neighbor-joining method. The four laccases used in this study are in red. Three fungal categories including basidiomycetes, ascomycetes and others are in blue, red and yellow, respectively. Accession numbers of fungal laccases in this analysis are in Supplementary Table S2.

**Figure S3. Enzyme purity verified by SDS-PAGE.** The full-length 10% SDS-PAGE gel of crude filtrate and purified DLac (a), TvLac (b), AbLac (c), and MtLac (d) protein were presented. Coomassie Blue staining was used for visualizing proteins on the gels. The “Crude” was commercial available laccase samples dialyzed against 20 mM Tris-HCl (pH 8.0) before purification by chromatography.

**Figure S4. All laccases used in this study were stable at a high protein concentration.** The stability of laccases incubated at 5 μg/mL during the pre-incubation process evaluated by enzyme activity assay. The enzyme activity of each laccase, including DLac, TvLac, AbLac, and MtLac, without solvent, was measured immediately and defined as 1-fold. The pre-incubation condition in this experiment was laccase incubated in Tris buffer without any organic solvent. After 1 hr of pre-incubation, the laccase activities were measured to evaluate their stability over time. No statistically significant differences were found (P > 0.05 in Student’s t-test) among these samples. Data were shown as mean ± SD from three independent repeats.

**Figure S5. Actual activities of fungal laccases in the organic-solvent pre-incubation experiments.** Actual DLac, TvLac, AbLac, and MtLac activity after pre-incubation with different organic solvents. Laccases were pre-incubated with 30% organic solvent, and after 1 hr of pre-incubation, enzyme activity was assayed with ABTS as substrate. The highly significant difference (**) was set at P < 0.001 in Student’s t-test, compared to their Tris buffer control. Data were shown as mean ± SD from three independent repeats.

**Figure S6. Actual activities of fungal laccases in organic solvent by using phenolic substrates.** Actual laccase activities were measured after acetone and ethanol pre-incubation, with 2,6-DMP (a) or guaiacol (b) used as a substrate. Purified laccases including DLac, TvLac, and MtLac were pre-incubated with acetone or ethanol for 1 hr before enzyme activity assay. n.d. = not determined. The highly significant difference (**) was set at P < 0.001 in Student’s t-test, compared to their Tris buffer control. Data were shown as mean ± SD from three independent repeats.

**References**

1 Chen, V. B. *et al.* MolProbity: all-atom structure validation for macromolecular crystallography. *Acta Crystallogr*. *D Biol*. *Crystallogr*. **66**, 12-21 (2010).
